# Predicting sex from brain rhythms with deep learning

**DOI:** 10.1038/s41598-018-21495-7

**Published:** 2018-02-15

**Authors:** Michel J. A. M. van Putten, Sebastian Olbrich, Martijn Arns

**Affiliations:** 1 Department of Clinical Neurophysiology, MIRA-Institute for Biomedical Technology and Technical Medicine, University of Twente & Medisch Spectrum Twente, Enschede, The Netherlands; 2 Department of Psychiatry, Psychotherapy, and Psychosomatics, Psychiatric Hospital, University of Zurich, Zurich, Switzerland; 3 Research Institute Brainclinics, Nijmegen & Dept. of Experimental Psychology, Utrecht University, Utrecht, The Netherlands

## Abstract

We have excellent skills to extract sex from visual assessment of human faces, but assessing sex from human brain rhythms seems impossible. Using deep convolutional neural networks, with unique potential to find subtle differences in apparent similar patterns, we explore if brain rhythms from either sex contain sex specific information. Here we show, in a ground truth scenario, that a deep neural net can predict sex from scalp electroencephalograms with an accuracy of >80% (***p*** < **10**^−**5**^), revealing that brain rhythms are sex specific. Further, we extracted sex-specific features from the deep net filter layers, showing that fast beta activity (20–25 Hz) and its spatial distribution is a main distinctive attribute. This demonstrates the ability of deep nets to detect features in spatiotemporal data unnoticed by visual assessment, and to assist in knowledge discovery. We anticipate that this approach may also be successfully applied to other specialties where spatiotemporal data is abundant, including neurology, cardiology and neuropsychology.

## Introduction

Identification of sex from visual assessment of biometric data, in particular the face, is an important part of social perception, a skill typically acquired in the first year after birth^1,2^. Male and female brains differ as well, not only functionally and anatomically^3–6^, but also in the likelihood for development of neuropsychiatric diseases and responses to treatment^7^. Brain rhythms are the electrophysiological signatures of brain function^8–10^, and scalp electroencephalogram (EEG) recordings in pathologies like postanoxic coma or seizures are very distinct from physiology^11–13^.

In neuropsychiatric conditions, the correlation between brain rhythms and pathology is much less clear, and various quantitative techniques have been proposed to extract relevant features, for instance in patients with attention-deficit hyperactivity disorder^14^ or depression^15,16^. Sex, however, cannot reliably be extracted from visual or quantitative assessment of EEG^9,13^, despite significant sex differences in the structural connectome of the human brain^17^.

For several decades, traditional machine learning techniques have been frequently applied to brain imaging data, including electroencephalography (EEG), with applications ranging from characterization of the EEG background pattern^18,19^ or quantification of focal or global ischaemia^20–22^ to detection of epileptiform discharges^23,24^ and diagnostics in depression^16^. Common to most of these techniques is the requirement for prior assumptions to guide extraction of particular features to be used for classification^25^. Examples include spectral features or correlations between EEG signals from different brain regions^26,27^. A limitation of these approaches is that unknown and potentially relevant features may not be included. Deep nets do not need prior extraction of such hand-made features, can learn from raw data^28–30^, and have potential to detect subtle differences in otherwise similar patterns^25,28^. Here, we report on sex prediction from human scalp EEG recordings using a deep convolutional neural network.

## Results

**Figure 1:**
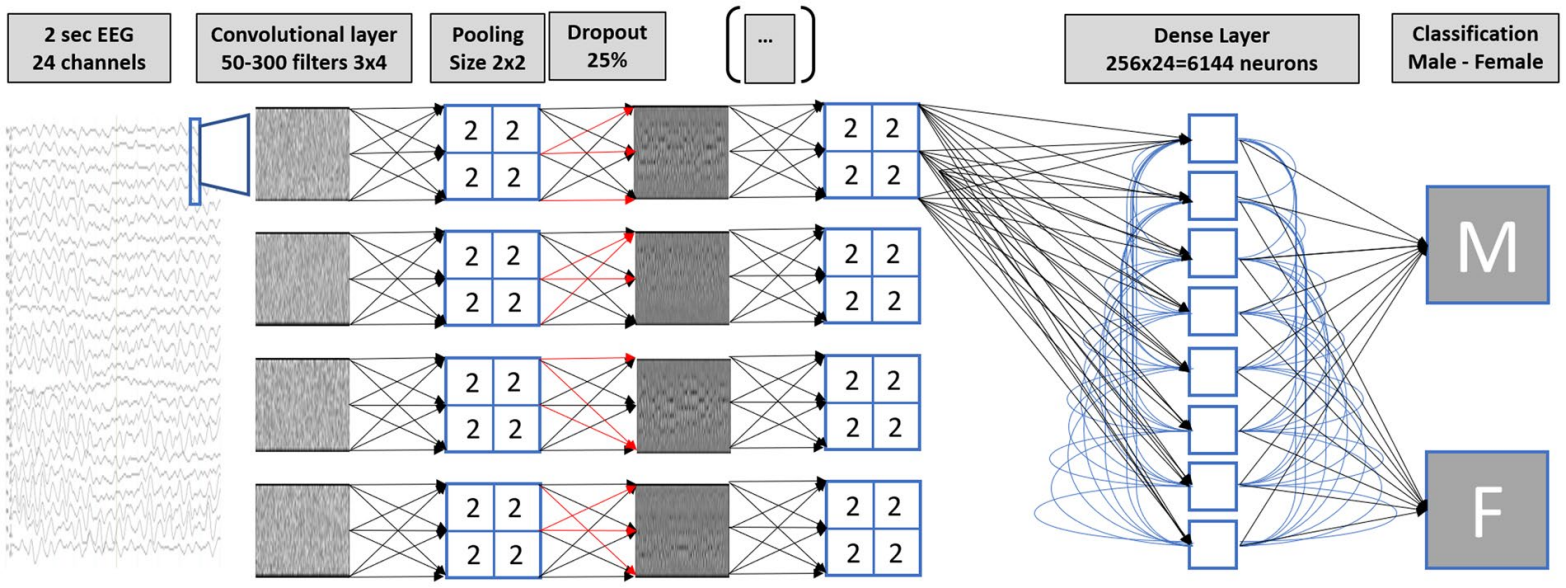
Architecture of the convolutional neural network. The input shape (2 second 24-channel EEG) has dimensions 256 (samples) × 24 (channels); the output of the net is dichotomous: 1 (male) or 0 (female). Stochastic optimization was realized using Adamax^51^ with learning rate = 0.002, β1 = 0.9, β2 = 0.999, ε = 10^8^ and decay = 0.0. As the loss function, the categorical cross-entropy was used. The total number of parameters was 9,051,902.

We used normative EEG data of 1308 subjects (mean age 43.38 (18.42 SD) yrs.; range 18–98 y; 47% males) recorded at different laboratories. We implemented a convolutional neural network in Python 3.6 using the Keras wrapper with a Tensorflow 1.0 backend on a CUDA-enabled NVIDIA GPU (GTX-1060), running in Windows 10 operating system. Figure 1 shows the global architecture (see Methods for more details).

**Figure 2:**
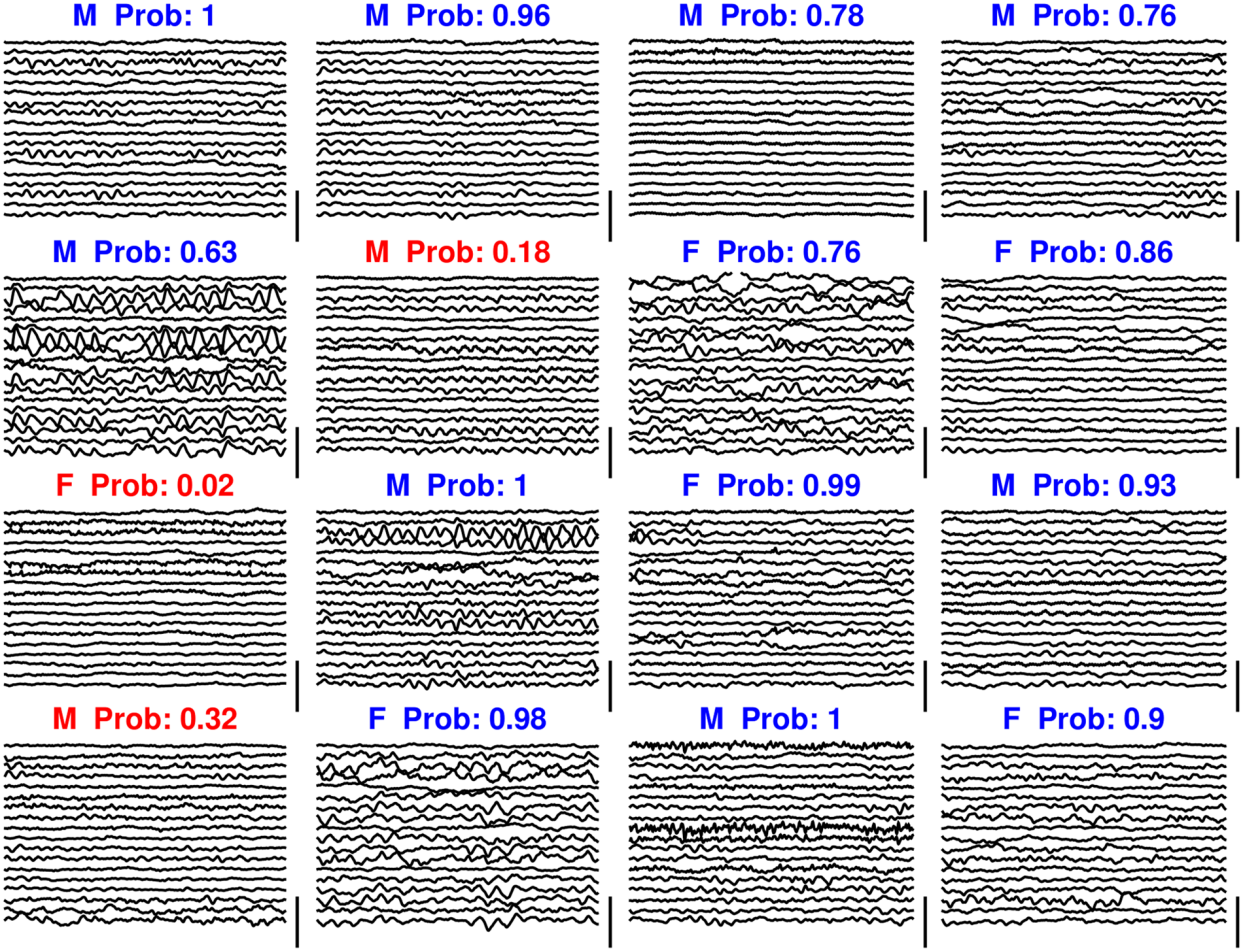
Examples of classification of raw scalp eyes-closed electroencephalograms (2 s epochs). Vertical bars are 70 μV. F = female, M = male. Prob: probability for the (true) class. In these 16 cases, three classifications (in red) were incorrect.

After training, accuracy was expressed as the percentage of correct classifications over all subjects, taking the mean probability of the 40 segments of 2 s each for each subject. We estimated statistical significance at p < 10^−5^ by Monte Carlo simulation, yielding a significance threshold for the classification accuracy of 63% (see methods for more details). We could subsequently predict male or female sex with an accuracy of 81%, far above the significance threshold of 63%. Examples of EEG epochs from the test set used for the classification are illustrated in Fig. 2. While spatiotemporal patterns differ, no distinct pattern for sex can be observed.

Using different hyperparameters, e.g. larger filter sizes (3 × 2 or 4 × 2 patches) or lower numbers of convolutional layers (four or three layers instead of 6 layers), resulted in inferior prediction accuracies of 61–69% and 63–74%, respectively. Although a myriad of possible networks can be constructed (as an example: evaluating 6 different layers, 10 different patch sizes and 10 different numbers of filters would result in 6 × 10^12^ distinctive networks), we restricted to similar network designs as stated above.

When the input matrices contained EEG from only the left or right hemisphere (each with 10 EEG channels, midline channels left out) with a consecutively slightly adjusted net architecture (i.e. halved input matrix with 256 × 10 channels and consecutive decrease of pooling along the y-axis), accuracies were 76% for the left hemisphere and 75% for the right hemisphere.

**Figure 3:**
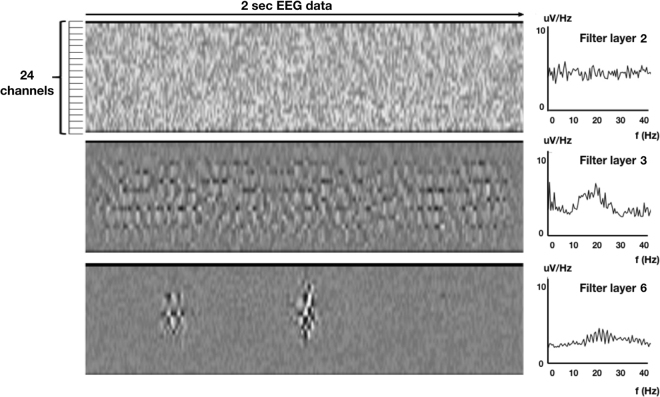
Panels left: The three filters generated input data with maximum activation for layer 2 (top), layer 3 (middle) and layer 6 (bottom), representing the features with most discriminative power between sexes. The Fourier Spectral analysis in the time domain (panels right, averaged for all 24 rows, similar to a traditional Fourier analysis approach of EEG data) displays that filters of layer 2 yield white noise without prominent features, while filters for layer 3 show a distinct peak in the beta range (12–25 Hz), similar to layer 6. In this example, the activation of layer 6 is mainly seen within rows (i.e. EEG-channels) 4–20, restricting the extracted feature to certain EEG-channels, providing additional spatial information.

As a second step, we were interested in the features the deep net learned to differentiate between males and females. When visualizing the filters of all six convolutional layers of the net using a procedure similar to the deep dream algorithm^31,32^, the first two filter layers produced data in the input space (i.e. as an artificially generated 2-sec EEG epoch) where spectral analysis with Fast Fourier Transformation (FFT) showed white noise (Fig. 3-left and right panel 1). Advancing deeper into the network, the consecutive filters (layers three to six) produced input data that reveal specific frequency features in the time domain when computing spectral analysis on each row, representing EEG-channel data (see Fig. 3-left panel 2). Since these artificially computed matrices reflect input patterns with the largest activation of the net, they can be regarded as surrogate EEG-segments, thus sharing similar frequency properties of original EEG data. The FFT analysis revealed that all deep layer filters > two show highest power peaks within the beta (12–25 Hz) frequency range (Fig. 3-right panel). The last layers (5 and 6), while also yielding mainly beta activity, restricted their focus on spatial patterns within the input space (Fig. 3-panels 3), thus showing sex-specific spatial differences in brain rhythms.

**Figure 4:**
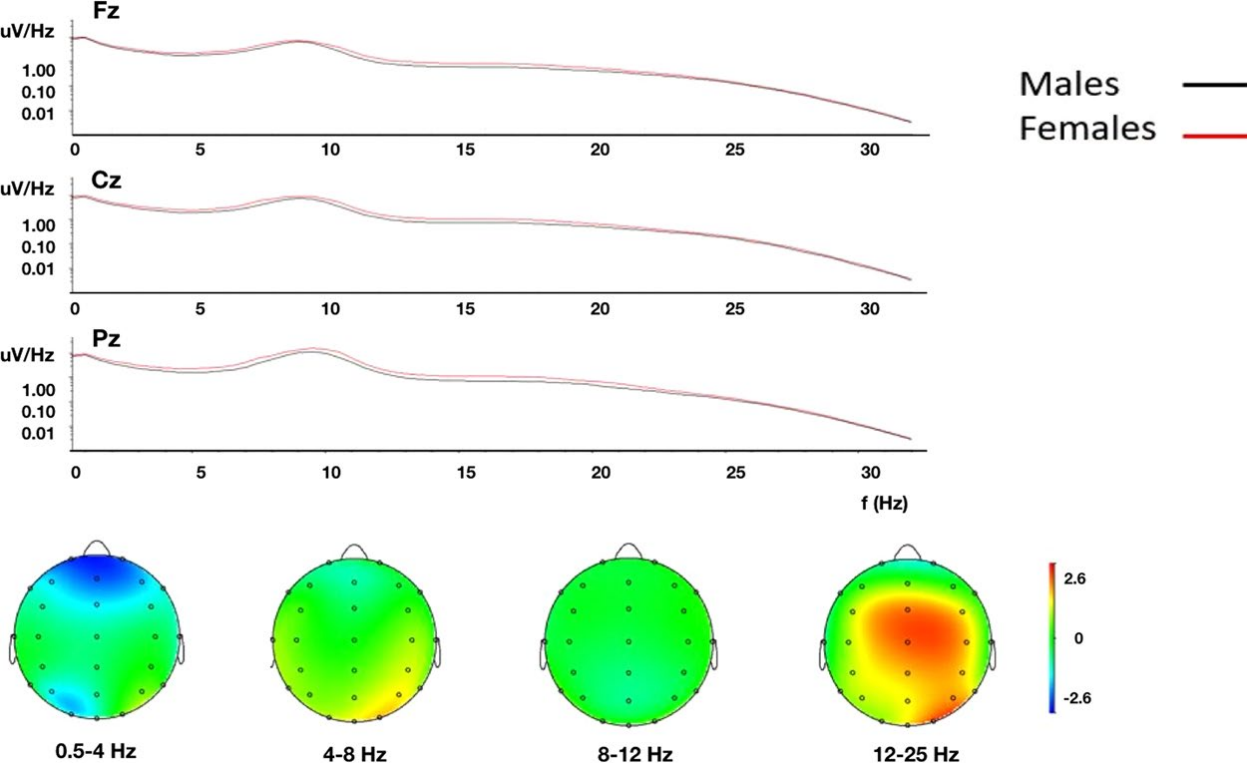
Top: Shown is the log-transformed amplitude spectrum in the frequency range 0.5–35 Hz from three electrode positions (Fz, Cz, and Pz). Power is slightly increased at all frequencies for females, most prominent in the beta band. Bottom: topoplots of the grand average (n = 1308) showing that differences in spectral content between males and females are mainly within the beta range (12–25 Hz; right). Colorbar at the right indicates z-scores.

Using this information of beta range activity being distinctive for sex classification, we went back to a traditional approach and extracted frequency power features from the EEG raw data. Exploiting the EEG-beta power from all subjects from all channels in a logistic regression model, we reached a classification accuracy of 70%, which is much lower than the achieved 81% of the deep net (Fig. 4).

## Discussion

We show that human scalp EEG recordings contain sex specific information that can be extracted with a deep convolutional network, reaching prediction accuracies better than 80%. To our knowledge, this is the first study to explore deep learning for sex classification. A prior study in a small sample (n = 40; 20 males), observed power differences in resting state EEG for the delta (0–4 Hz), theta (4–8 Hz), alpha 2 (10–12 Hz) and beta bands (13–25 Hz), but predictive accuracies were not reported^33^.

While not all details of the features used for classification by the deep net have been revealed, our data show that differences in brain rhythms between sexes are mainly in the beta frequency range (cf. Figs 3 and 4). Women are generally better at recognizing emotions and expressing themselves than men^34^, in part also reflected in differences in responses from the mu-rhythm as a presumed read-out of the mirror neuron system^35^, and modulations of beta activity during wakefulness have been associated with cognition and emotionally positive or negative tasks^36^. The discovery from the deep net that information in the beta-range differs between the sexes supports these observations. However, which particular spatiotemporal characteristics of the beta-rhythm differentiate remains enigmatic, and was not further explored.

Our CNN was motivated by the architecture used for image classification with ImageNet^37^. The input matrices of the EEG data were shaped as a two-dimensional input array, comparable to a two-dimensional image. We decided to use a 6-layer network since the input space matrix comprised of 256 × 24 data points. Hence, a pooling approach with halving data points after each layer will result in 4 data points left along the x-axis. Further convolution on a 4 × 1 matrix should not bring in additional discriminative power for the network. Since an increase of data points in the input space (e.g. by using a higher sampling rate with e.g. 256 or 500 Hz or larger epoch lengths) did increase the computational needs above the available resources, we restricted to the maximal depth of six layers. No further attempts were made to compare different architectures. The main goal of our work was to demonstrate that sex specific information is contained in scalp EEG recordings that can be extracted with deep learning. Future studies should investigate further optimizations of this approach e.g. by varying montages, filter settings and network architectures. Although EEG recordings where obtained from different laboratories, this is not a significant limitation of our work, since all data was recorded with a standardized platform, the same amplifiers and across-site consistency; test-retest reliability of this methodology has been published before^38,39^.

Deep learning for analysis of EEG patterns has been applied to other studies with resting state EEG. For instance, a deep net learned discriminative features between imagining music or listening to music^40^ or rhythm perception^41^. Differentiation between early stage Creutzfeldt-Jakob disease and other forms of rapid progressive dementias with deep learning achieved a sensitivity of 92% at specificity of 89%^42^. Classification of sleep stages using deep nets performed on par with human sleep experts^43^. Detection of interictal epileptiform discharges is another promising application for deep nets, and may soon become a standard clinical tool to assist in the diagnostic process in epilepsy^44,45^.

In sum, brain rhythms contain sex specific information, and deep convolutional networks can extract features from time series beyond traditional approaches, including visual assessment. Deep nets might also substitute or complement human guided feature extraction and knowledge discovery in other specialties where spatiotemporal data are ubiquitous, including clinical neurophysiology, cardiology, intensive care medicine, psychiatry and neuropsychology. This approach may also find application to differentiate response characteristics to drugs^7^, with promise to contribute to diagnostic and prognostic applications in personalized medicine^46^.

## Methods

### Normative data

EEG data were obtained from six different laboratories that were extracted from the Brain Resource International Database (New York, Rhode Island, Nijmegen, London, Adelaide and Sydney). All participants were adults (mean age 43.38 (18.42 SD) y; range 18–98 y; 47% males). Exclusion criteria were a personal or family history of mental illness, brain injury, neurological disorder, serious medical condition, drug/alcohol addiction, first-degree relative with bipolar disorder, schizophrenia, or genetic disorder. Institutional review board approval was obtained for all sites and informed consent from all subjects. All methods were performed in accordance with the relevant guidelines and regulations. IRB approval was obtained for all sites (Nijmegen: Commissie Mensgebonden Onderzoek, Regio Arnhem-Nijmegen; CMO-nr: 2002/008).

### EEG recordings

EEG recordings were performed using a standardized methodology and platform (Brain Resource Ltd., Australia) for which full details have been published elsewhere^7,47^ as have the results of the across-site consistency and reliability of this methodology^38,39^.

Participants were seated in a sound and light attenuated room, controlled at an ambient temperature of 22 °C. EEG data were acquired from 26 channels: Fp1, Fp2, F7, F3, Fz, F4, F8, FC3, FCz, FC4, T3, C3, Cz, C4, T4, CP3, CPz, CP4, T5, P3, Pz, P4, T6, O1, Oz and O2 (Quikcap; NuAmps; 10–20 electrode international system, sampling frequency 500 Hz). Data were referenced to averaged mastoids with a ground at AFz. Horizontal eye movements were recorded with electrodes placed 1.5 cm lateral to the outer canthus of each eye. Vertical eye movements were recorded with electrodes placed 3 mm above the middle of the left eyebrow and 1.5 cm below the middle of the left bottom eyelid. Skin-electrode impedance was kept <5 kOhm. A low pass filter with an attenuation of 40 dB per decade above 100 Hz was employed prior to digitization. EEG data was recorded for two minutes with eyes open (EO) with the participant asked to fixate on a red dot on the screen. Two minutes with eyes closed (EC) were obtained while the participant was instructed to remain relaxed. Data were EOG-corrected using a regression-based technique similar to that used by Gratton, Coles and Donchin^48^ and stored in EDF format^49^.

EEG data was down-sampled to 128 Hz and subsequently band-pass filtered between 0.5–25 Hz. EEG reference was kept unchanged (averaged mastoids) and 24 channels were kept (Fp1, Fp2, F7, F3, Fz, F4, F8, FC3, FCz, FC4, T3, C3, Cz, C4, T4, CP3, CPz, CP4, T5, P3, Pz, P4, T6, O1) while two were removed (O2 and Oz) to achieve low numbers in the prime decomposition of the matrix (3 × 2 × 2 × 2) to later be able to perform a maximum of pooling operations. Filtering was obtained with a first order Butterworth minimum phase distortion filter.

### Deep Net Architecture

The architecture of the deep net was inspired by deep convolutional nets that have been designed for image classification^37^. The input matrix for the net was a 24 (EEG-channels) × 256 (2 s × 128 Hz) matrix. For the filter sizes of the convolutional layers, we used minimal windows of 2 × 2 patches. The number of filters decreased from 300 within the first and second layer to 50 within all other layers. Activation was done using a rectified linear unit. A pooling function was applied before using a dropout function for the first four convolutional layers. The final classification was obtained by applying a dense layer with a softmax activation, resulting in a probability p for male or female sex. The various layers are summarized in Table 1.

**Table 1:** Layers of the CNN. The input matrix for the net was a 24 (EEG-channels) × 256 (2 s × 128 Hz) matrix. RELU = rectified linear unit.

Layer type	activation	# of filters	filter size	dropout rate
1. Convolutional	RELU	100	3 × 3	
2. Pooling			2 × 2	
3. Dropout				25%
4. Convolutional		100	3 × 3	
5. Pooling			2 × 2	
6. Dropout				25%
7. Convolutional		300	2 × 3	
8. Pooling			2 × 2	
9. Dropout				25%
10. Convolutional		300	1 × 7	
11. Pooling			1 × 2	
12. Dropout				25%
13. Convolutional		100	1 × 3	
14. Convolutional		100	1 × 3	
15. Dense	softmax			

### Training and testing

We trained the neural network using 40 non-overlapping EEG segments of 2 s duration with eyes closed from every subject. In total, EEGs from 1000 adults were used for the training set (40 epochs × 1000 subjects = 40000 epochs of 2 s with 47% being males). Each segment received one-hot label array, indicating a male or a female. Training was done with a batch size of 70 for 150 runs, meaning all 40000 epochs were presented to the network 150 times in chunks of 70 segments.

Training and testing the accuracy of the data was done on large separate, independent datasets, therefore cross validation was deemed not necessary. The independent test set comprised 308 cases (49% males, 40 segments from each subject × 308 subjects = 12320 samples of 2 s). Classification by the final layer of the network was binary (male (1) or female (0)). Within training, accuracy was computed after each run for all segments of the training set and for the test set. Training was finished after a) accuracy within the training set reached 100% or b) the loss function of the training set did not further decrease or c) 150 runs were finished. Final classification was dichotomous, by taking the mean probability of the 40 segments of 2 s each for each subject; if p > 0.5, the EEG was classified as male.

### Visualizing deep layers

The procedure we used to visualize which features of the input data are mainly used by the CNN is similar to a technique called “deep-dreaming” and has been described elsewhere in more detail^31,32,50^. The essence of the method is that the network is activated “top-down”, meaning that from a desired output (e.g. 1 = male) from the last layer, the connections of the trained network are activated toward the input layer. The activity of the first layer, which normally receives the input matrices (i.e. the raw EEG data), then can be seen as an artificially generated input pattern that most likely would produce the desired output. During this process, the filter layers in between the input and the output are activated, representing archetypal features of the desired output.

We generated artificial input patterns by retrograde ascending of the gradients in the trained network model, repeating this for all filters of all layers and sorting the generated data for the input space by the highest loss (i.e. the maximum activation of a specific filter in a particular layer^32^).

### Estimating significance

First, we randomly assigned sex to each subject in the test set (n = 308), using the prior sex distribution (47% males). To set the p-value for statistical significance at p < 10^−5^, we performed 100,000 simulations in Matlab. The best classification accuracy reached was 63%, which was subsequently considered the significance threshold.

### Spectral features

Power spectrum was estimated using a Fast Fourier Transform using Welch’s method with half overlapping epochs of 10 s, as implemented in Brain Vision Analyzer 2.1.0 (Gilching, Germany).
